# Investigation of the Potential Mechanism of *Alpinia officinarum* Hance in Improving Type 2 Diabetes Mellitus Based on Network Pharmacology and Molecular Docking

**DOI:** 10.1155/2023/4934711

**Published:** 2023-02-09

**Authors:** Xuguang Zhang, Xiangyi Li, Hailong Li, Mingyan Zhou, Yuxin Zhang, Weiyong Lai, Xiuwen Zheng, Feihu Bai, Junqing Zhang

**Affiliations:** ^1^Hainan Provincial Key Laboratory for Research and Development of Tropical Herbs, Haikou Key Laboratory of Li Nationality Medicine, School of Pharmacy, Hainan Medical University, Haikou, China; ^2^The Gastroenterology Clinical Medical Center of Hainan Province, Department of Gastroenterology, The Second Affiliated Hospital of Hainan Medical University, Haikou, China

## Abstract

**Objective:**

We used network pharmacology, molecular docking, and cellular analysis to explore the pharmacodynamic components and action mechanism of *Alpinia officinarum* Hance (*A. officinarum*) in improving type 2 diabetes mellitus (T2DM).

**Methods:**

The protein-protein interaction (PPI) network, Gene Ontology (GO), and Kyoto Encyclopedia of Genes and Genomes (KEGG) enrichment analyses were performed to predict the potential targets and mechanism of *A. officinarum* toward improving T2DM. The first 9 core targets and potential active compounds were docked using Discovery Studio 2019. Finally, IR-HepG2 cells and qPCR were applied to determine the mRNA expression of the top 6 core targets of the PPI network.

**Results:**

A total of 29 active ingredients and 607 targets of *A. officinarum* were obtained. T2DM-related targets overlapped with 176 targets. The core targets of the PPI network were identified as AKT serine/threonine kinase 1 (AKT1), an activator of transcription 3 (STAT3), tumor necrosis factor (TNF), tumor protein p53 (TP53), SRC proto-oncogene, nonreceptor tyrosine kinase (SRC), epidermal growth factor receptor (EGFR), albumin (ALB), mitogen-activated protein kinase 1 (MAPK1), and peroxisome proliferator-activated receptor gamma (PPARG). *A. officinarum* performs an antidiabetic role via the AGE-RAGE signaling pathway, the HIF-1 signaling pathway, the PI3K-AKT signaling pathway, and others, according to GO and KEGG enrichment analyses. Molecular docking revealed that the binding ability of diarylheptanoid active components in *A. officinarum* to core target protein was higher than that of flavonoids. The cell experiments confirmed that the *A. officinarum* extracts improved the glucose uptake of IR-HepG2 cells and *AKT* expression while inhibiting the *STAT3*, *TNF*, *TP53*, *SRC*, and *EGFR* mRNA expression.

**Conclusion:**

*A. officinarum* Hance improves T2DM by acting on numerous components, multiple targets, and several pathways. Our results lay the groundwork for the subsequent research and broaden the clinical application of *A. officinarum* Hance.

## 1. Introduction

Type 2 diabetes mellitus (T2DM) has gradually become a global epidemic disease, influencing global public health, and its incidence is rising. According to recent figures issued by the International Diabetes Federation, 783 million adults are estimated to develop diabetes by 2045 [1]. Nevertheless, the pathogenesis of T2DM is intricate, including genetics, lifestyle, environment, and unidentified factors [2]. It is characterized by persistently high blood sugar levels due to insulin deficiency or insulin resistance. Currently, the treatment for T2DM is typically diet-based, supplemented with the use of oral drugs or insulin injections for patients who cannot regulate their blood glucose levels through diet management alone [3, 4]. However, varying degrees of gastrointestinal events and other risks may still occur with the therapy of hypoglycemic Western medication, resulting in drug withdrawal [5]. Exploring a therapeutic drug with mild action, better efficacy, and higher safety is therefore critical for the treatment of T2DM patients.

*Alpinia officinarum* Hance (*A. officinarum*), also known as smaller galangal, is a dry rhizome of *A. officinarum*, a member of the Ginger Family Alpinia from Guangdong, Hainan, Guangxi, and Taiwan. The main functions are to warm the stomach and stop vomiting, disperse cold, and relieve pain [6, 7]. *A. officinarum* is commonly used in China and Europe to improve blood sugar levels, stomach pain, swelling, and cold [8]. It has been widely used in food flavors and flavorings [9, 10]. Modern pharmacological studies have shown that *A. officinarum* and its extract can alleviate T2DM by promoting glucose metabolism and lowering the blood sugar levels [11–13]. However, the potential mechanism of *A. officinarum* against diabetes is unknown.

Owing to its characteristics of integrity and systematization, as well as based on the principle of traditional Chinese medicine (TCM) holistic view and syndrome differentiation, network pharmacology has gradually become one of the important means of predicting the mechanism of TCM treatment for various diseases [14, 15]. The molecular docking method employs a personal computer to simulate the binding conformation of ligand and receptor macromolecules and to predict their binding energies [16]. Therefore, accumulating studies have applied network pharmacology and molecular docking to clarify the pharmacodynamic basis and molecular mechanism of TCM/drugs in treating diseases [17].

The primary active components, prospective targets, and pharmacological mechanisms of *A. officinarum* were identified using a method based on network pharmacology by integrating molecular docking in this study. IR-HepG2 cells and the qPCR method were employed to validate the proposed molecular mechanism. These findings provide some predicted and inferred evidence for elucidating *A. officinarum's* material basis and the action mechanism in improving T2DM.

## 2. Materials and Methods

### 2.1. Network Pharmacology

#### 2.1.1. Collection of Active Compounds and Targets of *A. officinarum*

The active compounds of *A. officinarum* were screened through the Traditional Chinese Medicine Systems Pharmacology Database and Analysis Platform (TCMSP) database (https://old.tcmsp-e.com/tcmsp.php) [18], the Bioinformatics Analysis Tool for Molecular Mechanism of Traditional Chinese Medicine (BATMAN-TCM) platform database (https://bionet.ncpsb.org/batman-tcm/) [19], and the Encyclopedia of Traditional Chinese Medicine (ETCM) database (https://www.tcmip.cn/ETCM/index.php/Home/Index/index.html) [20]. Furthermore, the compounds were supplemented by reviewing the related relevant articles [21–23]. After merging the results to remove the duplicates, the pharmacokinetic parameters of the collected components were evaluated using the Swiss ADME (https://www.swissadme.ch/) database, which could predict the components based on their chemical structures. *In vivo *pharmacokinetics-related information includes gastrointestinal (GI) absorption and Lipinski's 5 rules as the most important indicators [24]. Based on the output results, the active components of *A. officinarum* were screened preliminarily.

TCMSP, BATMAN-TCM, ETCM, and the SwissTargetPrediction database (https://www.swisstargetprediction.ch/) [25] were referred to search for targets corresponding to the active components of *A. officinarum*. Subsequently, the target protein was transformed into the corresponding target gene through the UniProt database (https://www.uniprot.org/) [26]. The database of *A. officinarum* compounds and their targets was constructed. Finally, the resultant components and the targets were employed to construct a component-target (C-T) network diagram of *A. officinarum* by using the Cytoscape 3.8.2 software (https://apps.cytoscape.org) [27].

#### 2.1.2. Identification of T2DM-Related Targets

Using “Type 2 diabetes mellitus” as the keyword, T2DM-related genes were screened from the GeneCards database (https://www.genecards.org) [28], PharmaGKB database (https://www.pharmgkb.org/) [29], Therapeutic Target Database (TTD, https://db.idrblab.net/ttd/) [30], DrugBank database (https://www.drugbank.ca/) [31], and the Online Mendelian Inheritance in Man database (OMIM, https://www.omim.org/) [32]. Then, the obtained data were merged, the duplicate entries were removed, and these targets were identified as potential targets for *A. officinarum* to improve T2DM. Finally, the results were visualized using the EVenn network tool (https://www.ehbio.com/test/venn/#/) [33].

#### 2.1.3. Prediction of the Potential Targets for Improving T2DM with *A. officinarum*

In order to acquire the *A. officinarum* common target in the amelioration of T2DM, the EVenn network tool was applied to construct the Venn diagram of the *A. officinarum* target genes and the T2DM-target genes, and the overlaps were considered as the potential targets of *A. officinarum* toward improving T2DM.

#### 2.1.4. Protein-Protein Interaction (PPI) Analysis

PPI data for the intersection targets of *A. officinarum* and T2DM were obtained from the STRING database (https://string-db.org) [34]. The protein interaction data were gathered and imported into the Cytoscape 3.8.2 software (https://apps.cytoscape.org) to create the PPI network diagram. Then, the core targets of the network were screened by applying the degree (D), betweenness centrality (BC), and closeness centrality (CC) in the CytoNCA analysis [35, 36].

#### 2.1.5. Gene Ontology (GO) and the Kyoto Encyclopedia of Genes and Genomes (KEGG) Pathway Enrichment Analyses

GO functional analysis and the KEGG pathway enrichment analysis were performed on the potential targets by using the Metascape database (https://metascape.org/gp/index.html#/) [37]. *P* < 0.05 was used as the screening condition, and the top 20 signaling pathways and the top 10 GO terms were shortlisted. Moreover, the GO terms were classified into 3 aspects, including cellular component (CC), molecular function (MF), and biological process (BP). Finally, the bioinformatics software platform (https://www.bioinformatics.com.cn/login/) was applied to visualize the ultimate outcomes.

#### 2.1.6. Construction of the Network of “Component-Target-Pathways”

The “component-target-pathway” (C-T-P) network of *A. officinarum* to improve T2DM was constructed by the Cytoscape 3.8.2 software. The nodes of the network were mainly composed of *A. officinarum*, active components, potential targets, the top 20 signaling pathways, and their targets screened by the KEGG pathway enrichment analysis. Moreover, the core active component was further determined based on the degree value of the node.

#### 2.1.7. Molecular Docking

The structures of the 29 components were painted in the Discovery Studio 2019 [38]. The crystal structures of AKT1 (PDB ID: 6HHI), STAT3 (PDB ID: 6NJS), EGFR (PDB ID: 41Z5), PPARG (PDB ID: 1I7I), ALB (PDB ID: 1E7H), SRC (PDB ID: 4U5J), TP53 (PDB ID: 6GGA), TNF (PDB ID: 6X83), and MAPK1 (PDB ID: 6G54) were downloaded from the RCSB database (https://www.RCSB.org). The proteins were added to the Discovery Studio software to remove water and ligands from the environment to define their binding sites. After preparing the receptors and ligands, the (LibDock) docking mode was selected to perform semiflexible and fast molecular docking, with the conformation set to “BEST” and the remaining parameters set to default. After the docking was complete, the successfully docked compounds were sorted in descending order by score. The CDOCKER mode was selected to better understand the ligand-binding mechanism, and the protein receptor was docked with the ligands and positive drugs with the highest LibDock scores for flexible molecular docking. The “Pose Cluster Radius” was set to 0.5, and the other parameters were left alone. After the docking was complete, the binding force of the successfully docked compounds was expressed as “CDOCKER ENERGY.”

### 2.2. *In Vitro* Analyses

#### 2.2.1. Preparation of *A. officinarum* Extract

*A. officinarum* rhizomes were procured in Haikou, China, in July 2013. Professor Niankai Zeng performed the authentication, and a sample (No. 20130716) was deposited in the Medicinal Chemistry Laboratory of Hainan Medical University. The dried and crushed *A. officinarum* (1.5 kg) was heated and refluxed with 80% ethanol thrice for 1 h, each time. The solution was filtered and concentrated at room temperature (200 g) to obtain the *A. officinarum* extract (AOE). The components of the AOE were identified using high-performance liquid chromatography (HPLC), and the representative chromatogram of phytochemical compounds in *Alpinia officinarum* extract is shown in Figure S1.

#### 2.2.2. Cell Culture and Treatment

HepG2 cells were procured from the Zhong Qiao Xin Zhou Biotechnology (Shanghai, China) and cultured in high-glucose Dulbecco's modified essential medium (DMEM, Gibco, United States) supplemented with 10% fetal bovine serum (FBS, Gibco) and 1% penicillin-streptomycin (Biosharp, Anhui, China) under 37°C in a 5% CO_2_ cell incubator. The cells in the log phase were used for subsequent experiments. To investigate the effect of AOE on IR, the cells from the past methods were grown in 50 mM glucose for 48 h to establish an IR-HepG2 cell model [39]. In addition, HepG2 cells were classified into 3 groups: control group (5.5 mM DMEM), model group (50 mM DMEM), and AOE group (50 g/mL AOE + 50 mM DMEM).

#### 2.2.3. Determination of Cell Viability

Cell viability was measured by using the Cell Counting Kit-8 assay (CCK-8 assay; Beyotime, Shanghai, China). Then, the HepG2 cells were seeded at the concentration of 1 × 10^4^ cells/well in a 96-well plate and cultured in the DMEM medium supplemented with 10% CS. After 24 h of cell adhesion, the cells were treated with different concentrations of AOE (1–200 *µ*g/mL) for 48 h. Then, 10 *μ*L of the CCK8 solution was added to each well and the plate was incubated at 37°C for 1 h. The absorbance was measured at 450 nm by using the Spectra Max plus Automatic Plate Reader (Molecular Devices, Sunnyvale, CA, USA).

#### 2.2.4. Glucose Uptake Analysis

The glucose uptake assay was performed using the fluorescence probe (2-NBDG, Invitrogen, USA), and the HepG2 cells were cultured in a 6-well plate until the cells reached >70% confluence. After IR induction and AOE treatment, the cells were stimulated in a low-glucose DMEM containing 100 nmol/L insulin for 30 min, followed by treatment with 25 mM of 2-NBDG/well for 30 min. Next, the cells were washed with cold PBS until they became colorless. The fluorescence intensity was then read with the Gemini XPS fluorescence microplate (Molecular Devices) reader with the excitation and emission wavelengths of 485 and 535 nm, respectively.

#### 2.2.5. Quantitative Polymerase Chain Reaction (qPCR) Analysis

The mRNA expression of *AKT*, *STAT3*, *TNF*, *TP53*, *SRC*, and *EGFR* was determined by qPCR. HepG2 cells were seeded in a 6-well plate until the cells reached >70% confluence. After IR induction and AOE treatment, respectively, total RNA was extracted from HepG2 cells by using the Eastep® Super Total RNA Extraction kit (Shanghai Detection and Biological Products Co., Ltd., China). The cDNA template was then reverse transcribed using the HiScript® II Q RT Super Mix for qPCR Kit under the following PCR amplification reaction conditions: initial activation of thermostart DNA polymerase for 5 min at 95°C, the second step of 40 cycles (i.e., 95°C for 10 s, 60°C for 20 s, and 72°C for 20 s). Then, the melting curve phase was followed, and finally, the amplification primers were obtained. All primer sequences were synthesized by Sangon Biotech (Shanghai, China): *AKT* (Forward, 5′-TGACCATGAACGAGTTTGAGTA-3′; Reverse, 5′-GAGGATCTTCATGGCGTAGTAG-3′),*STAT3* (Forward, 5′-GGACTGAGCATCGAGCA-3′; Reverse, 5′-GCCAGACCCAGAAGGAG-3′),*TNF* (Forward, 5′-GGAAAGGACACCATGAGC-3′; Reverse, 5′-CCACGATCAGGAAGGAGA-3′),*TP53* (Forward, 5′-CCAGATGAAGCTCCCAGA-3′; Reverse, 5′-GGGAAGGGACAGAAGATGA-3′), SRC (Forward, 5′-GTACGTGGAGCGGATGAAC-3′; Reverse, 5′-GAGCCAGCCCAAAGTCC-3′),*EGFR* (Forward, 5′-GCCTGAAAACAGGACGGA-3′; Reverse, 5′-GAGGGAGCGTAATCCCAAG-3′), and *GAPDH* (Forward, 5′-CCTTCCGTGTCCCCACT-3′; Reverse, 5′-GCCTGCTTCACCACCTTC-3′). The relative expression of each gene was analyzed by the 2^−ΔΔCt^ method, and all genes were normalized to their GAPDH levels.

### 2.3. Statistical Analysis

The data were expressed as the mean ± SD (*n* ≥ 3). Differences between the samples were analyzed by one-way ANOVA with GraphPad Prism 8.0.1. *P* < 0.05 was considered to indicate statistical significance.

## 3. Results

### 3.1. Identification of the Potential Targets of *A. officinarum* Improves T2DM

**Figure 1:**
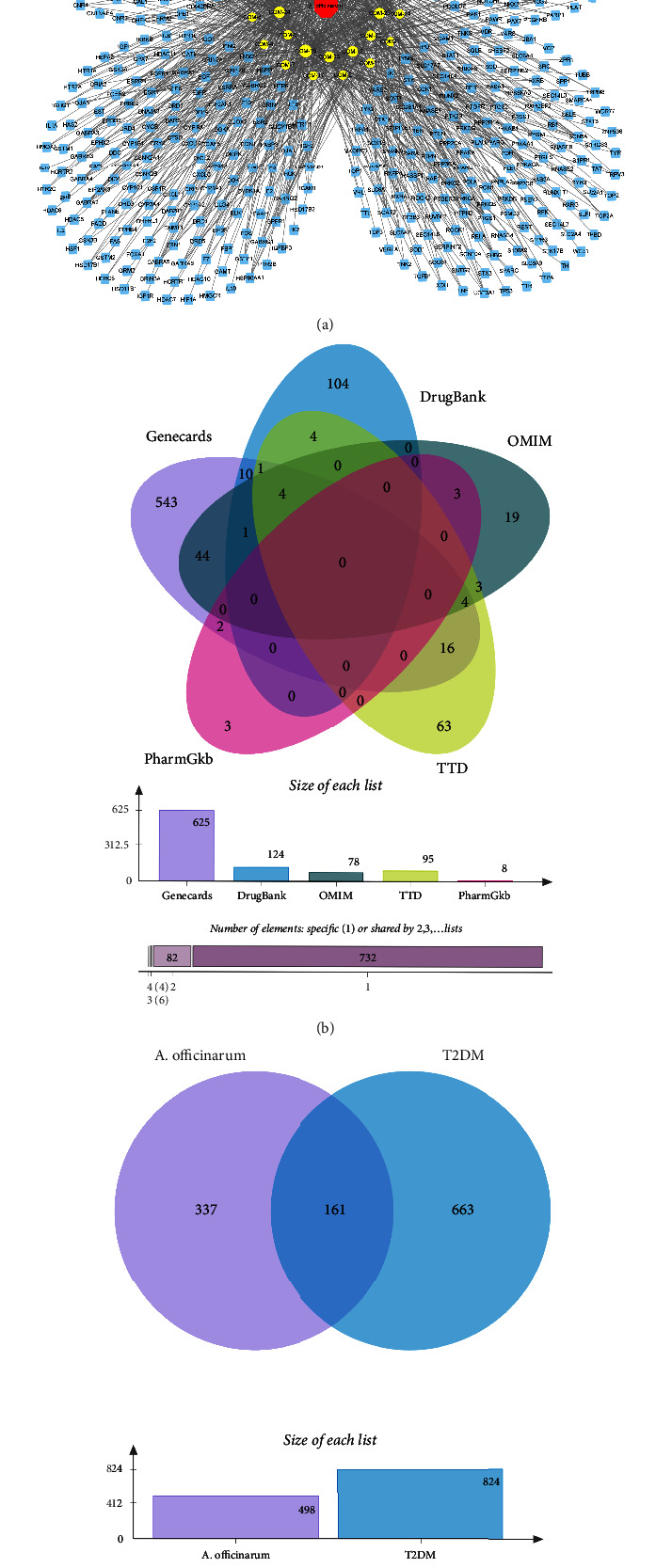
The potential targets of *A. officinarum* improve T2DM. (a) The component-target network diagram of *A. officinarum*, blue nodes: targets of *A. officinarum*, yellow nodes: components of *A. officinarum*; (b) the Venn diagram of T2DM-relative targets; (c) the Venn diagram of T2DM- and *A. officinarum*-related targets.

The screening of a multisource database yielded 29 active components of *A. officinarum*. As shown in Table 1, the majority of the 29 compounds were flavonoids and diarylheptanoids, volatile oils, and sterols, which were regarded as the active substances of *A. officinarum* in the treatment of T2DM. After eliminating the duplicates, a total of 607 potential targets of *A. officinarum* were obtained. The C-T network graph also includes 637 nodes (29 active ingredients, 607 related targets, and 1 plant) and 1,168 edges (Figure 1(a)).

A total of 625, 8, 95, 125, and 78 potential T2DM therapeutic targets were screened using the GeneCards, PharmGKB, TTD, DrugBank, and OMIM databases, respectively. After removing the duplicates, 824 targets were considered potential T2DM therapeutic targets (Figure 1(b)). Subsequently, the intersection of *A. officinarum* and T2DM targets was obtained, yielding a total of 176 targets that may be regarded as *A. officinarum*-potential targets for enhancing T2DM (Figure 1(c)).

### 3.2. PPI Network of Potential Targets

**Figure 2:**
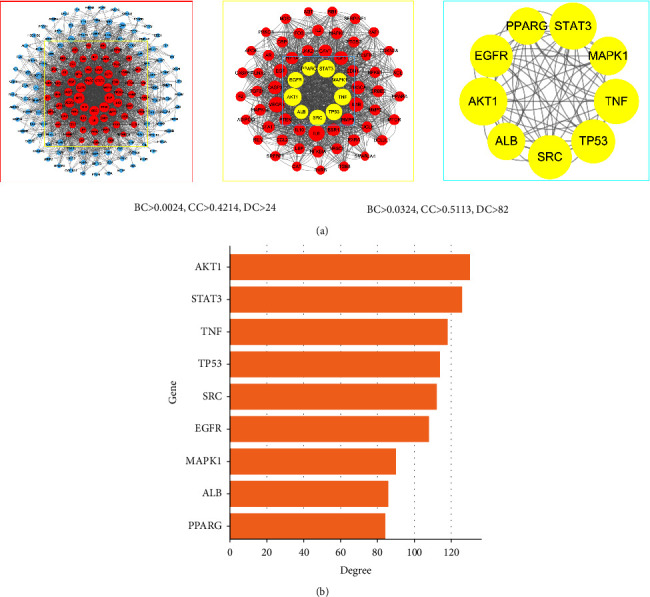
PPI network analysis of potential targets of *A. officinarum* and T2DM. (a) Protein-protein interaction (PPI) network, DC: degree; BC: betweenness centrality; CC: closeness centrality; (b) degree values of the top 9 target proteins.

A PPI network was built and evaluated using the Cytoscape 3.8.2 software after predicting reliable interaction information for 176 potential targets from the STRING database. After hiding the unconnected nodes, a PPI network consisting of 170 nodes and 2,814 edges was obtained (Figure 2(a)). The CytoNCA app was applied to analyze the centrality of each node in the PPI network, namely, the degree, betweenness centrality, and closeness centrality, to further investigate the key targets in the PPI network. Therefore, according to the results of CytoNCA, a total of 9 key nodes were further screened out, which were considered to be the core targets for *A. officinarum* against T2DM, including AKT1 (AKT serine/threonine kinase 1), STAT3 (signal transducer and activator of transcription 3), TNF (tumor necrosis factor), TP53 (tumor protein P53), SRC (SRC proto-oncogene, non-receptor tyrosine kinase), EGFR (epidermal growth factor receptor), ALB (albumin), MAPK1 (mitogen-activated protein kinase 1), and PPARG (peroxisome proliferator-activated receptor gamma). Furthermore, the magnitude of the degree value indicates the importance of the node, and Figure 2(b) shows the degree values of the 9 core target proteins. It was discovered that AKT1 had the highest degree value. As a result, AKT1 was determined to be the most central target in the PPI network.

### 3.3. GO and KEGG Enrichment Analyses

**Figure 3:**
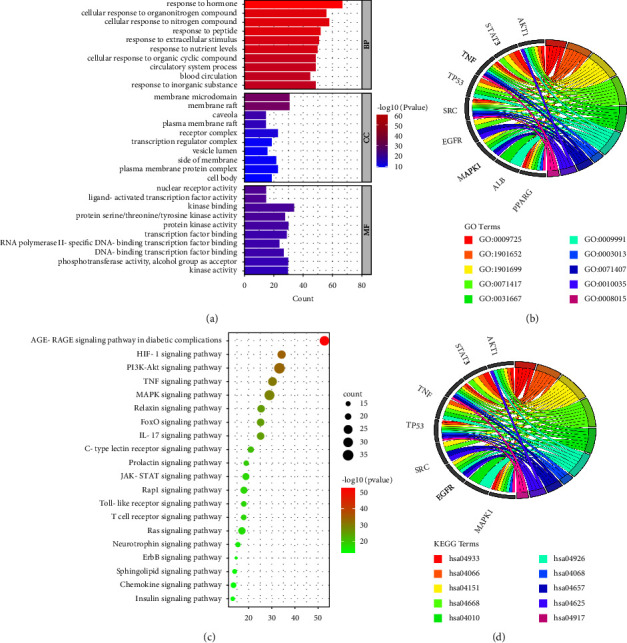
GO and KEGG enrichment analyses of the potential targets of *A. officinarum* and T2DM. (a) GO enrichment analysis of the targets of *A. officinarum*; (b) analysis of KEGG enrichment in 20 pathways as the targets of *A. officinarum*; (c) the chord diagram of GO terms and core targets; (d) the chord diagram of the KEGG core pathways and core targets.

GO enrichment analysis was performed to explore the functions of 176 targets shared by *A. officinarum* and T2DM, yielding 2,631 GO items, including 2,324 BPs, 118 CCs, and 189 MFs terms. Considering *P* < 0.05 as the screening criterion, the top 10 BPs indicated that the common genes of *A. officinarum* and T2DM were mainly involved in hormone response, peptide response, organic nitrogen compound response, and circulatory system process (Figure 3(a)). The GO keywords enriched for MFs in the enrichment analysis of CCs including membrane raft, membrane microdomain, caveola, and plasma membrane raft were mainly ligand-activated transcription factor activity, nuclear receptor activity, kinase binding, and transcription factor binding. Meanwhile, we constructed a chord diagram of the core targets and the top 10 GO terms to determine whether they are involved in the top 10 GO terms. As shown in Figure 3(b), the core targets were all involved in the top 10 GO terms, with AKT1, TNF, and SRC being implicated the most.

The KEGG pathway enrichment analysis revealed a total of 81 pathways. Considering the enrichment value as the reference basis, we determined the top 20 pathways as the main drug pathways in Figure 3(c). The relative enrichment analysis revealed the following signaling pathways: AGE-RAGE, HIF-1, PI3K-AKT, TNF, MAPK, and various other hormone transduction signaling pathways. Furthermore, to investigate whether core targets are involved in the top 10 signaling pathways, we constructed a chord diagram of the core targets and the top 10 signaling pathways. As shown in Figure 3(d), the core targets were all involved in the top 10 KEGG terms. AKT1 and MAPK1 are involved in the largest number of the top 10 signaling pathways. The abovementioned results indicated that *A. officinarum* exerts anti-T2DM efficacy in multiple biological processes and signaling pathways.

### 3.4. Construction of the Network of “Component-Target-Pathways”

**Figure 4:**
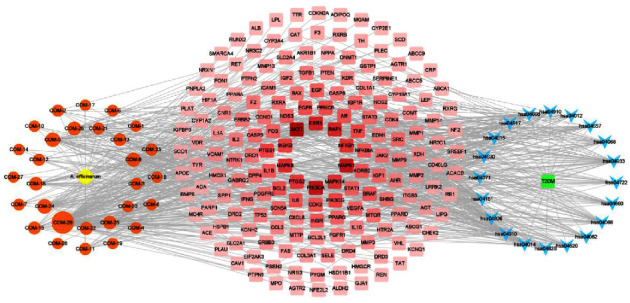
The “component-target-pathways” network of *A. officinarum* for T2DM. Blue nodes: the KEGG pathway of the GO-term identifier; red nodes: the potential targets of *A. officinarum* improve T2DM, and the redder the color of the node, the higher the degree value of the node; orange node: components of *A. officinarum*, and the larger the node, the greater the degree value of the node; yellow node: *A. officinarum*; green node: T2DM.

The “component-target-pathway” network was constructed based on the top 20 significant KEGG signaling pathways and their corresponding targets to further investigate the molecular mechanism of *A. officinarum* to improve T2DM (Figure 4). This network included 225 nodes and 841 edges (1 plant, 29 compounds, 176 targets, 20 pathways, and 1 disease). The size of the compound node (orange node) in the network was proportional to its topological score, and the higher the degree value, the larger the node shape, the lower the degree value, and the smaller the node shape. As a result, the top 6 active ingredients (quercetin, kaempferol, 5-hydroxy-7-(4″-hydroxy-3″-methoxy-phenyl)-1-phenyl-3-heptanone, 7-(4″-hydroxy-3″-methoxyphenyl)-1-phenyl-hept-4-en-3-one, galangin, and medicarpin) were screened based on the size of the network nodes, which were considered to be the main active compounds to be beneficial in the treatment of *A. officinarum*. According to the network analysis, 29 active components of *A. officinarum* might regulate multiple key proteins and act on multiple pathways to help alleviate T2DM.

## 4. Results of Molecular Docking

**Figure 5:**
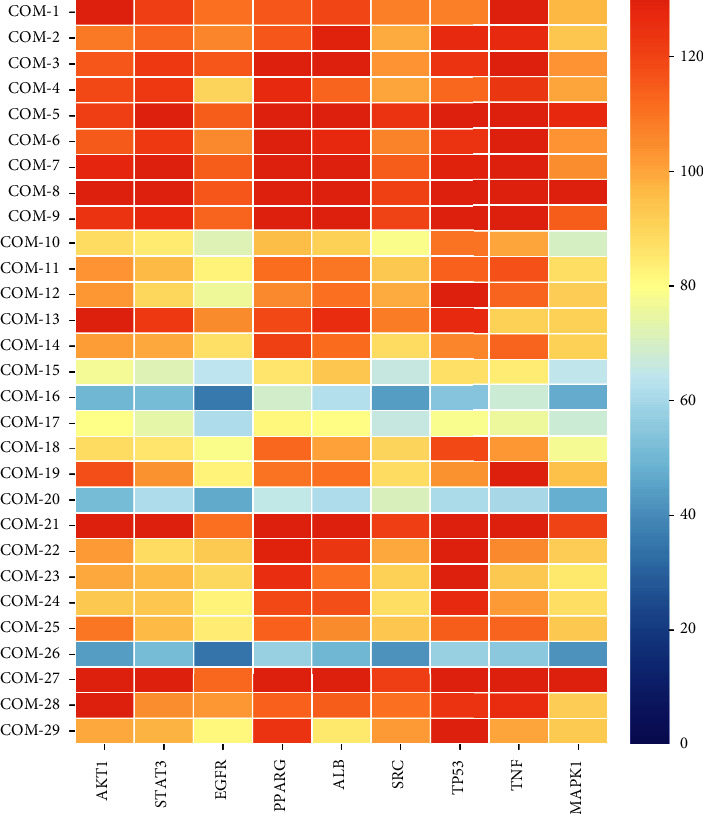
Heat map of LibDock scores for active compounds in *A. officinarum* and the core target proteins. The *X*-axis represents the key targets; *Y*-axis represents the bioactive compounds. The redder the color, the higher the LibDock score.

To better explain the binding activity between the active compounds of *A. officinarum* and core target proteins, molecular docking was applied to simulate 9 core targets (i.e., AKT, STAT3, TNF, TP53, SRC, EGFR, ALB, MAPK1, and PPARG) and 29 active compounds. Figure 4 depicts the statistical results of the LibDock scores of the docking targets. The higher the value in the figure, the darker the color, indicating a better binding effect between the compound and the receptor protein (Figure 5). The LibDock scores of all core components and the key targets in this analysis were all >50 (Table S1), indicating that *A. officinarum* active components exhibited a high biological affinity for potential target molecules and the potential to exert a high pharmacodynamic activity. The strongest binding force to the 9 core target proteins is 5 diarylheptanoids, including hexahydrocurcumin (COM-21), octahydrocurcumin (COM-27), 1,7-diphenyl-5-hydroxy-3-heptanone (COM-3), 5-hydroxy-7-(4″-hydroxy-3″-methoxy-phenyl)-1-phenyl-3-heptanone (COM-5), and 5R-hydroxy-7-(4-hydroxy-3-methoxyphenyl)-1-(4-hydroxyphenyl)-3-heptanone (COM-8). Furthermore, 9 diarylheptanoids (COM-3-9, 21, 27) exhibited the highest binding scores to core targets, all >100, out of the 29 active components. Second, the binding scores of 7 flavonoids (i.e., COM-2, 12, 18, 22, 23, 24, and 29) and the core targets were all >80. As a result, this indicated that the principal medicinal components of *A. officinarum* are diarylheptanoids and flavonoids, with diarylheptanoids being more active.

**Figure 6:**
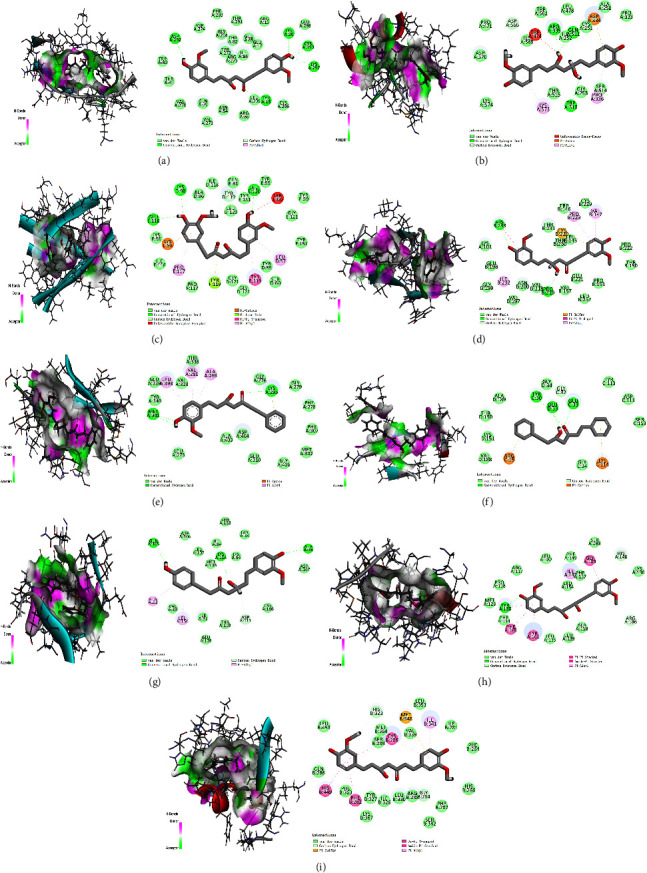
The docked complexes of 9 target proteins along with their strongest binding compounds. (a) AKT1-hexahydrocurcumin, (b) STAT3-octahydrocurcumin, (c) TNF-hexahydrocurcumin, (d) TP53-hexahydrocurcumin, (e) SRC-5-hydroxy-7-(4″-hydroxy-3″-methoxy-phenyl)-1-phenyl-3-heptanone, (f) EGFR-1,7-diphenyl-5-hydroxy-3-heptanone, (g) MAPK1-5R-hydroxy-7-(4-hydroxy-3-methoxyphenyl)-1-(4-hydroxyphenyl)-3-heptanone, (h) ALB-octahydrocurcumin, and (i) PPARG-hexahydrocurcumin.

To better understand the ligand-binding mechanism of *A. officinarum* active compounds, we selected the compounds with the highest ligand-binding scores from each core protein and docked them again in the CDOCKER mode.  Figure 6 shows that the active ingredient in *A. officinarum* binds to the core protein primarily carbon-hydrogen bond, conventional hydrogen bond, van der Waals, and others. To compare the molecular docking results, we selected resveratrol, napabucasin, thalidomide, 5-fluorouracil, bosutinib, celecoxib, ulixertinib, warfarin, and rosiglitazone as positive drugs with their corresponding proteins [40–48]. The active ingredient binds to AKT1, STAT3, TNF, MAPK1, ALB, and PPARG were found to be more effective than the positive drug, while binding to TP53, SRC, and EGFR was found to be slightly weaker (Table 2). This observation suggested that *A. officinarum* has a beneficial effect in the treatment of IR.

Overall, the molecular docking results were consistent with the network pharmacology results, indicating that *A. officinarum* exerted an antidiabetic effect by using numerous components, multiple targets, and several pathways.

### 4.1. *In Vitro* Study

#### 4.1.1. Effect of AOE on HepG2 Cell Viability

**Figure 7:**
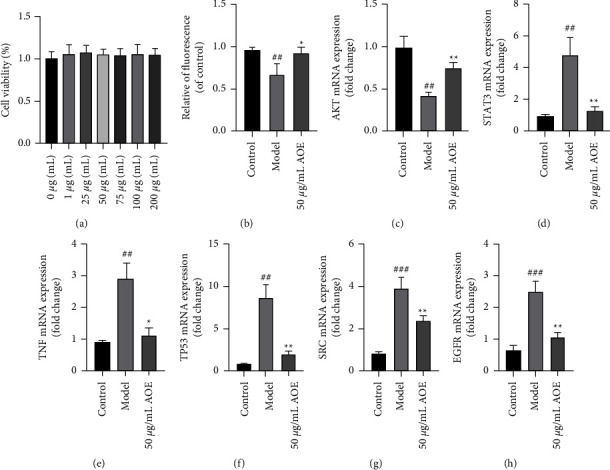
The effects of AOE on IR-HepG2 cells. (a) Cell viability; (b) quantitative fluorescence determined by the extent of glucose uptake; (c) the mRNA expression of AKT; (d) the mRNA expression of STAT3; (e) the mRNA expression of TNF; (f) the mRNA expression of TP53; (g) the mRNA expression of SRC; (h) the mRNA expression of EGFR. All values are expressed as the mean ± SD (*n* ≥ 3). ^*∗*^*P* < 0.05, ^*∗∗*^*P* < 0.01, when compared with the model control group; ^##^*P* < 0.01 and ^###^*P* < 0.001 when compared with the control group.

We analyzed the viability of HepG2 cells at different AOE concentrations to determine the AOE concentrations that did not alter cell viability. As shown in Figure 7(a), the cell viability of each group did not change significantly when compared to that of the normal control group treated with AOE at different drug concentrations for 48 h. Therefore, the results showed that AOE at concentrations of 1–200 *μ*g/mL had no obvious toxic effect on the HepG2 cells. Therefore, a dose of 50 *μ*g/mL of AOE was selected for subsequent analyses.

#### 4.1.2. Effect of AOE on Glucose Uptake in IR-HepG2 Cells

To explore the effect of AOE on IR-HepG2 cells, 50 mM glucose was used to induce IR, and intracellular glucose uptake was assessed by using the 2-NBDG method. The glucose uptake level of 3T3-L1 adipocytes was significantly decreased after 48 h of 50 mM glucose induction (*P* < 0.001) (Figure 7(b)), indicating that the IR model was successfully established. However, the results indicate that 50 *μ*M AOE might reverse the high glucose-induced decline caused by glucose uptake (*P* < 0.05).

#### 4.1.3. Effects of AOE on the mRNA Expression in IR-HepG2 Cells

The first six-core targets (i.e., AKT, STAT3, TNF, TP53, SRC, and EGFR) were determined by qPCR to validate the core targets predicted by network pharmacology. As shown in Figures 7(c)–7(h), the model group significantly suppressed the AKT mRNA expression when compared to the control group, indicating the occurrence of insulin signaling disorder, that is, insulin resistance. Furthermore, 50 ug/mL AOE may significantly increase the mRNA expression of AKT, promoting insulin signal transduction. The STAT3, TP53, and TNF mRNA expression were significantly decreased under high-glucose treatment but significantly increased after 48 h of AOE treatment. These results suggest that AOE can improve insulin resistance by lowering inflammation. SRC and EGFR are both involved in angiogenesis, and in the experiment, the mRNA expressions of SRC and EGFR in the model group were significantly decreased, which AOE significantly reversed. This finding suggests that AOE could improve angiogenesis and alleviate T2DM complications. Furthermore, these results were consistent with those predicted by network pharmacology.

## 5. Discussions

In this study, a total of 29 active ingredients of *A. officinarum* were obtained, including quercetin, kaempferol, galangin, medicarpin, 5-hydroxy-1,7-diphenyl-3-heptanone, octahydrocurcumin, 5-hydroxy-7-(4″-hydroxy-3″-methoxy-phenyl)-1-phenyl-3-heptanone, 7-(4″-hydroxy-3″-methoxyphenyl)-1-phenyl-hept-4-en-3-one, among others (Table 1). According to the type of ingredients, the 29 compounds were mainly flavonoids, diarylheptanoids, volatile oils, and sterols. Galangin, kaempferol, 5-hydroxy-7-(4″-hydroxy-3″-methoxy-phenyl)-1-phenyl-3-heptanone, 7-(4″-hydroxy-3″-methoxyphenyl)-1-phenyl-hept-4-en-3-one, and 5-hydroxy-1,7-diphenyl-3-heptanone are often used as the principal component indices of *A. officinarum* fingerprint [49]. Three components of *A. officinarum*, quercetin, kaempferol, and galangin, have been shown to improve T2DM. Quercetin enhances glucose metabolism and insulin resistance and protects *β* cells from oxidation-induced apoptosis [50, 51]. In addition, quercetin has specific renal and liver protective properties [52, 53]. Kaempferol reduces insulin resistance by inhibiting the inflammatory response as well as improving glucose metabolism and hepatic gluconeogenesis [54–56]. Galangin is also considered to be a potential DPP-4 inhibitor, as it improves insulin-stimulated skeletal muscle glucose uptake and glucose levels as well as oxidative damage in fructose-fed rats [57, 58]. In T2DM, quercetin, kaempferol, and galangin have been demonstrated to exhibit significant antidiabetic effects.

We employed the molecular docking method to verify the binding fraction of each active component to the core target protein in order to further verify the relationship between the main active components of *A. officinarum* and the predicted targets. The results revealed that diarylheptanoids had the highest binding fraction in *A. officinarum* and that flavonoids also had higher binding fractions. Considering the low content of some diarylheptanoids in *A. officinarum*, we speculated that 5-hydroxy-7-(4″-hydroxy-3″-methoxy-phenyl)-1-phenyl-3-heptanone, 7-(4″-hydroxy-3″-methoxyphenyl)-1-phenyl-hept-4-en-3-one, and 1,7-diphenyl-5-hydroxy-3-heptanone are important active ingredients.

A total of 167 potential targets of *A. officinarum* were identified to improve T2DM in this investigation. According to GO enrichment analysis, *A. officinarum* mainly exerts its anti-T2DM effect via hormone response, peptide response, organic nitrogen compound response, and others. The KEGG enrichment analysis identified the AGE-RAGE signaling pathway, HIF-1 signaling pathway, PI3K/AKT signaling pathway, and TNF signaling pathway as important signaling pathways in the network. Past studies have demonstrated that the AGEs-RAGE signaling pathway is closely related to DM and its complications. If IR occurs, excessive glucose levels in the blood can cause nonenzymatic glycosylation of proteins, lipids, and DNA in the body, resulting in the formation of excessively advanced glycosylation end-products (AGEs), which are widely distributed throughout the body [59]. HIF-1, also known as hypoxia-inducible factor 1, is the key regulator of oxygen metabolism, and it regulates both glucose uptake and glycolysis [60]. The PI3K/AKT signaling pathway is a classic insulin signaling pathway that promotes glucose metabolism while inhibiting hepatic gluconeogenesis [61]. The TNF signaling pathway's essential protein is TNF-*α*, which is one of the major proinflammatory mediators and induces low-leveltissue-specific inflammation by activating IKK*β* and promoting the development of insulin resistance and the onset of T2DM [62]. These results suggest that *A. officinarum* ameliorates T2DM by promoting insulin signaling, improving inflammatory responses, and attenuating diabetic markers.

AKT1, STAT3, TNF, TP53, SRC, EGFR, PPARG, ALB, and MAPK1 were identified as the main targets after a topological analysis of the PPI network. The molecular docking results showed that the active components of *A. officinarum* exhibited good binding ability to the core target protein. More importantly, *in vitro* experiments revealed that AOE may significantly increase the AKT mRNA expression while inhibiting the STAT3, TNF, TP53, SRC, and EGFR mRNA expression. Phosphorylation of AKT1 by phosphoinositide 3-kinase (PI3K) promotes the translocation of GLUT4 to the cell surface, which regulates glucose uptake, and GSK3B regulates gluconeogenesis [63]. Phosphorylation of STAT3 has been found in studies to aggravate lung injury in mice with T2DM-associated pulmonary tuberculosis [64]. TNF induces insulin resistance in adipocytes by inhibiting insulin-induced IRS1 tyrosine phosphorylation and insulin-induced glucose uptake [65]. In STZ-treated and db/db mice, P53 inhibition ameliorates mitochondrial dysfunction, and glucose intolerance [66]. Excessive SRC activation produces reactive oxygen species and impairs the pancreatic *β*-cells metabolic-secretory coupling [67]. In type 2 diabetic mice, Choung et al. revealed that inhibiting the EGFR activity increased glucose tolerance and insulin sensitivity [68]. Therefore, the results of the *in vitro* experiments indicated that AOE improves insulin resistance in IR-hepG2 cells by regulating the abovementioned core targets, further demonstrating that *A. officinarum* exerts anti-T2DM effects through multiple target multipath ways.

This study has some limitations. First, only molecular docking and *in vitro* experimental validation were performed. Therefore, subsequent validation should be combined with animal models. Second, we only verified the first 6 core targets by qPCR. Therefore, other targets (such as PPARG and ALB) or specific pathways (such as PI3K/AKT signaling pathway) can be selected for further verification. We plan to pursue these gaps as a focus of our future work so as to provide more reliable data supporting the elucidation of the treatment of T2DM with *A. officinarum*.

## 6. Conclusions

The active ingredients of *A. officinarum* Hance were used for network pharmacological analysis and cell experimentation in this study, and the main active components, action targets, and main signaling pathways of *A. officinarum* Hance in the improvement of type 2 DM were predicted. The main targets were AKT1, STAT3, TNF, TP53, SRC, EGFR, ALB, MAPK1, and PPARG, and the core pathways were the AGE-RAGE signaling pathway, HIF-1 signaling pathway, PI3K-Akt signaling pathway, TNF signaling pathway, and MAPK signaling pathway. Furthermore, cell experiments revealed that AOE may increase glucose uptake and AKT mRNA expression, while inhibiting the STAT3, TNF, TP53, SRC, and EGFR mRNA expression. These results suggest that *A. officinarum* Hance plays a role in the amelioration of T2DM through a variety of components, multiple targets, and several pathways, providing sufficient reference and guidance for future experimental research and clinical applications of *A. officinarum* Hance.

## Figures and Tables

**Table 1:** The active components of *A. officinarum*.

IDs	Molecule names	Sources	MW	GI absorptions	Lipinski rules
COM-1	Sitosterol	TCMSP	414.71	Low	Yes
COM-2	(2S,3R)-2-(3,4-Dimethoxyphenyl)-5,7-dimethoxychroman-3-ol	TCMSP	346.37	High	Yes
COM-3	5-Hydroxy-1,7-diphenyl-3-heptanone	TCMSP	282.38	High	Yes
COM-4	1-Hydroxy-1,7-bis (4-hydroxy-3-methoxyphenyl)-6-heptene-3,5-dione	ETCM	386.4	High	Yes
COM-5	5-Hydroxy-7-(4″-hydroxy-3″-methoxy-phenyl)-1-phenyl-3-heptanone	PubMed	328.4	High	Yes
COM-6	5-Methoxy-1,7-diphenyl-3-heptanone	TCMSP	296.4	High	Yes
COM-7	5-Methoxy-7-(4″-hydroxyphenyl)-1-phenyl-3-heptanone	BATMAN-TCM	312.4	High	Yes
COM-8	5R-Hydroxy-7-(4-hydroxy-3-methoxyphenyl)-1-(4-hydroxyphenyl)-3-heptanone	PubMed	344.4	High	Yes
COM-9	7-(4″-Hydroxy-3″-methoxyphenyl)-1-phenyl-hept-4-en-3-one	PubMed	310.39	High	Yes
COM-10	7-Hydroxy-3,5-dimethoxyflavone	ETCM	298.29	High	Yes
COM-11	7-Methoxy-8-(2′-ethoxy-3′-hydroxy-3′-methybutyl) coumarin	TCMSP	292.33	High	Yes
COM-12	Azaleatin	ETCM	316.26	High	Yes
COM-13	Beta-sitosterol	TCMSP	414.71	Low	Yes
COM-14	Butyl-2-ethylhexyl phthalate	TCMSP	348.48	High	Yes
COM-15	Delta-cadinene	BATMAN-TCM	204.35	Low	Yes
COM-16	Eucalyptol	BATMAN-TCM	154.25	High	Yes
COM-17	Eugenol	BATMAN-TCM	164.2	High	Yes
COM-18	Galangin	ETCM, BATMAN-TCM, TCMSP	270.24	High	Yes
COM-19	Galanolactone	BATMAN-TCM	318.45	High	Yes
COM-20	Guaiacol	ETCM	124.14	High	Yes
COM-21	Hexahydrocurcumin	BATMAN-TCM	374.43	High	Yes
COM-22	Isorhamnetin	TCMSP	316.26	High	Yes
COM-23	Kaempferide	ETCM	300.26	High	Yes
COM-24	Kaempferol	BATMAN-TCM, TCMSP	286.24	High	Yes
COM-25	Medicarpin	BATMAN-TCM, TCMSP	270.28	High	Yes
COM-26	Methylcinnamate	BATMAN-TCM	162.19	High	Yes
COM-27	Octahydrocurcumin	BATMAN-TCM	376.44	High	Yes
COM-28	Clionasterol	TCMSP	414.71	Low	Yes
COM-29	Quercetin	ETCM, BATMAN-TCM, TCMSP	302.24	High	Yes

**Table 2:** Binding energy and interactions of potentially active compounds/positive drug and their four target proteins.

Target proteins	Compounds	CDOCKER energy	Interacting residues
AKT1	COM-21	−55.1829	TRP A:80, THR A:81, ASP A:292, ASP A:274, PHE A:293, GLY A:294, THR A:82, TYR A:272 ARG A:273, ILE A:84, GLU A: 85, GLU A: 17, LYS A:20, GLU A:298, CYS A:310, LYS A:297, TYR A:18, CYS A:296, LEU A:295, ARG A:86, ASN A:54 GLN A:79, VAL A:271, VAL A:270
	Resveratrol	−34.0981	TRP A:80, THR A:81, TNR A:82, ILE A:84, TYR A:18, ARG A:273, GLN A:79, ASP A:274, ASN A:54, TYR A:326, TYR A:272, VAL A:271, VAL A:270

STAT3	COM-27	−50.6181	ASP A:570, PRO A:471, ASP A:566, ILE A:569, TRP A:474, TRP A:562, ARG A:335, ILE A:252, LEU A:478, GLN A:511, CYS A:251, ASP A:334, ALA A:250, PRO A:333, SER A:514, PRO A:336, GLY A:253, TRP A:510, THR A:515, LYS A:573, LYS A:574
	Napabucasin	−27.1109	ASP A:334, THR A:515, LYS A:573 ILE A:569, ARG A:335, ASP A:570, ASP A:566, PRO A:471, MET A:470, ILE A:467, CYS A:468, HIS A:332, PRO A:333

TNF	COM-21	−48.307	GLU E:116, LYS D:98, ALA D:96, ILE D:118, TYR D:119, LEU D:120, TYR E:151, LEU E:120, TYR E:59, SER E:60, TYR F:59, TYR F:151, LEU D:57, TYR D:59, SER D:60, TYR E:119, GLY D:122, GLY D:121, TYR F:119, PRO F:117, PRO E:117, ILE E:118, LYS E:98, LYS F:98
	Thalidomide	−32.7244	LEU D:120, TYR D:59, GLY D:121, TYR E:119, LEU D:57, TYR E:59, LEU E:57, LEU F:57, GLY E:122, TYR F:59, TYR F:151, GLY E:121, LEU E:120, TYR F:119, GLY F:121, SER F:60, LEU F:120, TYR D:151, GLN D:61, TYR D:119, SER D:60

TP53	COM-21	−13.3682	HIS B:233, THR A:231, CYS B:220, THR B:230, LEU B:145, TRP B:146, PRO B:223, CYS B:229, VAL B:147, PRO B:222, THR B:150, PRO B:151, GLU B:221, LEU B:257, VAL B:157, PRO B:219, VAL B:218, ASN B:200, VAL B:197, ILE B:232, GLY B:199, GLU B:198, LYS A:101
	5-Fluorouracil	−19.2804	LYS A:101, THR B:321, ILE B:232, VAL B:197, ASN B:200, GLU B:198, GLY B:199, HIS B:233

SRC	COM-5	−52.7039	MET A:341, TYR A:340, GLU A:339, LEU A:393, VAL A:32, VAL A:281, THR A:338, ALA A:293, GLY A:276, LYS A:295, GLY A:279, PHE A:278, PHE A:307, MET A:302, GLY A:406, GLU A:310, ASP A:404, ALA:403, LEU A:273
	Bosutinib	−54.0798	GLU A:510, ARG A:506, LYS A:442, THR A:508, PRO A:507, TYR A:511, ASP B:348, GLY B:352, GLN A:497, ARG A:500, GLU B:353, LYS B:351, LEU B:350, GLY B:355, LYS B:356, LYS B:458, ASN:532, GLU B:531, ARG B:359

EGFR	COM-3	−28.1563	ALA D:189, TYR C:30, GLY D:34, GLU D:33, GLY C:32, GLU C:33, TYR C:113, ASP C:111, SER C:153, LYS C:114, GLY C:34, ARG C:15, VAL D:188, LYS D:151, THR D:190
	Celecoxib	−35.4719	ILE D:31, GLY C:34, LYS C:114, GLY C:32, GLU C:33, ALA D:35, TYR C:30, GLY D:34, ARG C:15, GLU D:33, TYR D:30, LYS D:114, GLY D:32

MAPK1	COM-8	−53.4415	GLN A:105, ASP A:106, LEU A:107, MET A:108, LYS A:114, LYS A:54, ILE A:84, SER A:153, GLY A:34, ALA A:35, ASP A:167, CYS A:166, ASP A:111, THP A:110, GLU A:109, ILE A:31, LEU A:156, VAL A:39, ALA A:52
	Ulixertinib	−48.8575	CYS A:166, LYS A:54, LEU A:156, VAL A:39, ILE A:84, GLN A:105, ALA A:52, MET A:108, ASP A:106, THR A:110, LEU A:107, ILE A:31, LYS A:114, GLU A:109, ALA A:35, TYR A:36, GLY A:34, ASP A:167

ALB	COM-27	−53.6019	PRO A:118, ARG A:117, LEU A:182, PHE A:149, ILE A:142, LEU A:154, PHE A:157, GLY A:189, SER A:193, HIS A:146, LYS A:190, ARG A:186, ALA A:158, LEU A:139, LEU A:135, TYR A:161, PHE A:165, PHE A:134, TYR A:138, MET A:123
	Warfarin	−36.7731	LEU A:154, GLY A:189, MET A:123, PHE A:165, PHE A:134, TYR A:138, PRO A:118, LEU A:115, LEU A:182, ARG A:117, ARG A:186, ASP A:183, TYR A:161, ILE A:142, HIS A:146, PHE A:157

PPARG	COM-21	−49.206	GLN B:286, LEU B:453, HIS B:323, MET B:364, SER B:289, CYS B:285, VAL B:339, MET B:348, LEU B:353, ILE B:341, ILE B:281, PHE B:264, HIS B:266, PHE B:287, SER B:342, GLY B:284, ARG B:288, LEU B:330, ILE B:326, TYR B:327, LYS B:367, PHE B:282, PHE B:363, HIS B:449
	Rosiglitazone	−45.5298	HIS B:323, SER B:289, HIS B:449, ILE B:326, MET B:364, TYR B:327, LYS B:367, CYS B:285, ARG B:288, ILE B:341, GLY B:284, HIS B:266, ILE B:281, MET B:348, LEU B:353, VAL B:339, LEU B:340, PHE B:363, LEU B:330,GLN B:286

## Data Availability

The raw data supporting the conclusions of this article will be made available by the authors, without undue reservation.

## Supplementary Materials

Supplementary Materials Figure S1: the representative chromatogram of phytochemical compounds in *Alpinia officinarum* Extract. Table S1: LibDock score of active compounds to target proteins.
