# Supplementary material for: Investigation of the Potential Mechanism of Alpinia officinarum Hance in Improving Type 2 Diabetes Mellitus Based on Network Pharmacology and Molecular Docking
Source: Evid Based Complement Alternat Med. 2023 Feb 9;2023:4934711. doi: 10.1155/2023/4934711 (PMC9935802; doi:10.1155/2023/4934711)
Supplement: Supplementary Materials — Figure S1: the representative chromatogram of phytochemical compounds in Alpinia officinarum Extract. Table S1: LibDock score of active compounds to target proteins. [file 4934711.f1.docx]

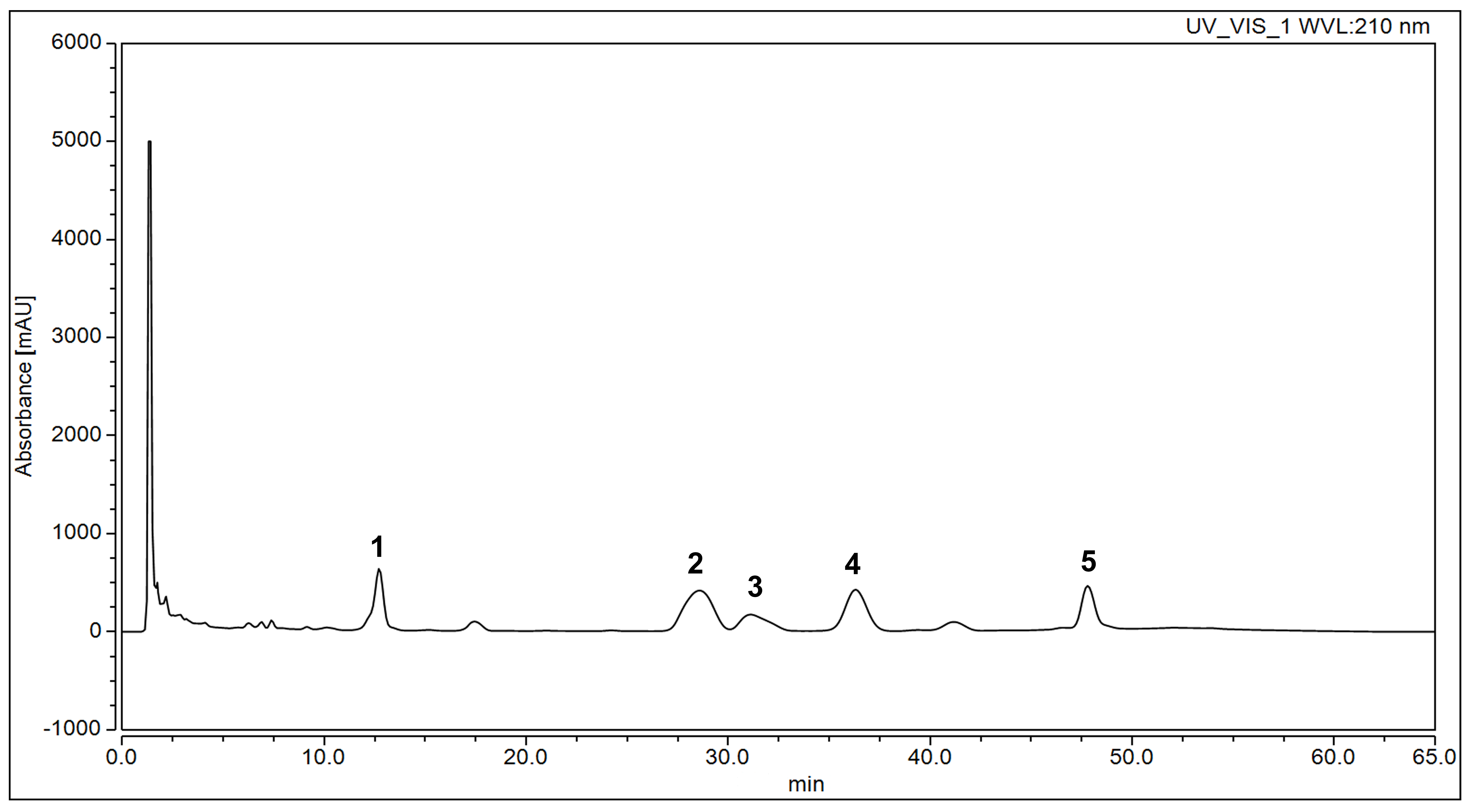


Figure S1: The representative chromatogram of phytochemical compounds in *Alpinia officinarum* Extract. (1) 5-Hydroxy-7-(4''-Hydroxy-3''-Methoxy-Phenyl)-1-Phenyl-3-Heptanone; (2) Galangin; (3) Galangin-3-methylether; (4) 7-(4''-Hydroxy-3''-Methoxyphenyl)-1-Phenyl-Hept-4-En-3-One; (5) 5-Hydroxy-1,7-diphenyl-3-heptanone.

Table S1: LibDock score of active compounds to target proteins.

| Component | AKT1 | STAT3 | EGFR | PPARG | ALB | SRC | TP53 | TNF | MAPK1 |
| --- | --- | --- | --- | --- | --- | --- | --- | --- | --- |
| COM-1 | 130.3 | 121.0 | 110.2 | 115.9 | 119.6 | 107.9 | 108.0 | 132.4 | 97.1 |
| COM-2 | 109.2 | 113.5 | 106.4 | 116.1 | 129.2 | 98.7 | 126.8 | 126.5 | 94.1 |
| COM-3 | 115.6 | 122.7 | 115.8 | 130.7 | 132.0 | 103.5 | 123.9 | 132.4 | 103.3 |
| COM-4 | 118.5 | 122.6 | 89.9 | 127.1 | 113.6 | 100.1 | 112.7 | 123.4 | 100.5 |
| COM-5 | 121.8 | 130.6 | 114.7 | 145.3 | 143.4 | 124.9 | 137.8 | 147.5 | 127.5 |
| COM-6 | 115.2 | 122.6 | 105.9 | 132.7 | 127.8 | 106.9 | 124.5 | 133.0 | 103.4 |
| COM-7 | 128.1 | 133.7 | 114.7 | 145.0 | 136.3 | 114.5 | 129.0 | 137.6 | 104.3 |
| COM-8 | 138.4 | 134.9 | 115.5 | 144.8 | 141.6 | 120.5 | 142.2 | 149.4 | 132.7 |
| COM-9 | 124.4 | 127.8 | 112.9 | 141.7 | 141.8 | 119.9 | 135.2 | 144.4 | 114.7 |
| COM-10 | 88.1 | 84.4 | 72.6 | 95.7 | 91.6 | 78.8 | 110.1 | 100.5 | 70.5 |
| COM-11 | 103.3 | 96.4 | 82.4 | 111.4 | 109.5 | 93.3 | 113.8 | 116.9 | 87.6 |
| COM-12 | 102.7 | 89.4 | 76.6 | 105.7 | 110.7 | 98.9 | 133.9 | 113.2 | 92.0 |
| COM-13 | 132.5 | 122.9 | 105.0 | 118.9 | 126.4 | 108.6 | 126.6 | 91.3 | 91.3 |
| COM-14 | 101.4 | 99.9 | 87.1 | 120.6 | 112.0 | 88.3 | 106.1 | 112.9 | 91.6 |
| COM-15 | 77.3 | 72.6 | 64.5 | 85.6 | 93.9 | 65.9 | 86.9 | 84.1 | 64.5 |
| COM-16 | 49.4 | 51.3 | 35.9 | 69.1 | 62.1 | 44.2 | 54.1 | 67.9 | 46.9 |
| COM-17 | 79.2 | 73.9 | 61.9 | 81.4 | 80.2 | 65.7 | 78.7 | 76.2 | 67.9 |
| COM-18 | 88.7 | 86.3 | 79.0 | 112.4 | 100.9 | 90.0 | 118.6 | 102.9 | 78.1 |
| COM-19 | 117.7 | 103.8 | 82.5 | 109.8 | 110.6 | 88.2 | 103.6 | 130.6 | 95.3 |
| COM-20 | 51.5 | 61.5 | 46.5 | 65.2 | 61.5 | 70.7 | 60.9 | 60.3 | 48.1 |
| COM-21 | 141.2 | 131.6 | 110.2 | 150.0 | 142.8 | 121.5 | 149.2 | 150.7 | 120.2 |
| COM-22 | 102.4 | 88.4 | 92.5 | 129.2 | 123.1 | 99.3 | 133.7 | 105.8 | 92.2 |
| COM-23 | 99.2 | 96.7 | 89.3 | 125.5 | 110.9 | 91.0 | 130.0 | 93.1 | 85.2 |
| COM-24 | 93.3 | 94.1 | 82.9 | 118.9 | 117.8 | 87.6 | 126.6 | 102.0 | 87.4 |
| COM-25 | 109.4 | 96.1 | 84.3 | 113.8 | 105.1 | 93.6 | 114.6 | 113.2 | 93.2 |
| COM-26 | 44.7 | 51.7 | 35.0 | 57.5 | 49.8 | 41.9 | 58.2 | 55.7 | 41.6 |
| COM-27 | 137.6 | 139.6 | 112.3 | 147.0 | 148.5 | 121.2 | 143.6 | 149.5 | 131.9 |
| COM-28 | 137.7 | 104.6 | 103.0 | 114.2 | 114.8 | 110.3 | 124.8 | 126.0 | 92.4 |
| COM-29 | 99.5 | 97.9 | 81.6 | 124.0 | 84.9 | 102.4 | 133.2 | 100.4 | 92.9 |
